# The Minimal Residual Disease in Non-Hodgkin's Lymphomas: From the Laboratory to the Clinical Practice

**DOI:** 10.3389/fonc.2019.00528

**Published:** 2019-06-26

**Authors:** Sara Galimberti, Elisa Genuardi, Francesco Mazziotta, Lorenzo Iovino, Fortunato Morabito, Susanna Grassi, Elena Ciabatti, Francesca Guerrini, Mario Petrini

**Affiliations:** ^1^Section of Hematology, Department of Clinical and Experimental Medicine, University of Pisa, Pisa, Italy; ^2^Department of Molecular Biotechnologies and Health Sciences, University of Torino, Turin, Italy; ^3^GeNOMEC School of Doctorate, University of Siena, Siena, Italy; ^4^Clinical and Translational Sciences School of Doctorate, University of Pisa, Pisa, Italy; ^5^Hematology Oncology Department, Augusta Victoria Hospital, East Jerusalem, Israel; ^6^Biotechnology Research Unit, Cosenza, Italy

**Keywords:** minimal residual disease, MRD, QT-PCR, PCR, digital PCR, NGS, NHL, lymphoma

## Abstract

Minimal residual disease (MRD) in non-Hodgkin's lymphomas (NHLs) still represents matter of interest and debate: indeed, the new available treatments offer higher rates of complete responses and MRD negativity than in the past, with a positive impact on the long-term survival. Furthermore, the introduction of more sensitive and accurate molecular techniques, such as digital PCR (ddPCR) and the next generation sequencing techniques (NGS), increased the possibility of identifying molecular targets to be followed after therapy (such as rearrangement of immunoglobulins, fusion genes, or mutations). This review focused on how molecular biology can help to detect MRD in different types of NHLs and how MRD can change the clinical practice in 2019. In follicular lymphoma (FL), contamination of the grafts and molecular disease persistence after transplantation represent a negative prognostic factors. The combination of Rituximab or Obinutuzumab with Bendamustine seems to be the most effective way to clear MRD in FL patients receiving chemo-immunotherapy (further studies are in progress), and also ^90^Yttrium-Ibritumomab-Tiuxetan offers a deep clearance of molecular disease. Finally, molecular MRD can further stratify PET-negative cases, with subjects both PET- and MRD-negative presenting the best outcome. In aggressive lymphomas, MRD has a relevant prognostic power and can represent the platform for immunotherapy (such as CAR-T). In diffuse large B-cell lymphoma (DLBCL), the assessment of MRD in the plasma (where cell-free DNA and exosomes circulate) seems to be more predictive than the bone marrow analysis or peripheral blood mononuclear cells. Finally, NGS technologies could be more useful than the classical “patient allele-specific PCR” because they can identify any possible clone emerging during the treatment or follow-up, even if different from that identified at diagnosis, thus predicting relapse. After all, the present available molecular approaches can move MRD from the bench side to the clinical practice.

## MRD in Non-Hodgkin's Lymphomas: an Overwiew

Today, physicians set as their goal the implementation and delivery of a “patient-tailored treatment” which has been known as “Precision Medicine;” unfortunately, even if several different biological and clinical factors have been assessed and considered as prognostic in different hematological malignancies, only few of them help to lead the therapeutic strategy, and the discovery of further prognostic tools still represents an unmet clinical need.

The first score system used for stratifying outcome in Non-Hodgkin's lymphomas (NHLs) was published in the New England Journal of Medicine in 1993 by the researchers taking part to the “International Non-Hodgkin's Lymphoma Prognostic Factors Project.” This project, involving more than 2,000 patients treated with chemotherapy containing doxorubicin in 16 institutions and cooperative groups in the USA, Europe, and Canada, gave origin to the “International Prognostic Index” (IPI) and to the “age-adjusted IPI” (aaIPI). IPI, based on age older than 60 years, disease stage III/IV, elevated lactate dehydrogenase, performance status ≥2, and more than one extra-nodal site involved, was able to identify four different groups with predicted 5 years OS of 73, 51, 43, and 26%, respectively. aaIPI, based on tumor stage, lactate dehydrogenase level, and performance status was also able to allocate patients into four risk groups with predicted 5 years OS rates of 83, 69, 46, and 32% (1).

More recently, other risk scores have been developed, and are now available and used at diagnosis to forecast the patients' outcome: FLIPI (2) and FLIPI2 (3) in follicular lymphoma (FL), R-IPI in diffuse large B-cell lymphoma (DLBCL) (4), MIPI in mantle cell lymphoma (MCL) (5), and MALT-IPI in the mucosa-associated lymphoid tissue (MALT) lymphoma (6).

All these scores are applied before treatment, and none of them is dynamic, except for the occurrence of progression within 24 months from diagnosis (the POD24), a “late” parameter which seems to be a valid prognostic factor in FL (7).

Furthermore, even if prognostic, none of these variables are commonly used for leading treatment (stop, prolong therapy, adopt a pre-emptive strategy, or consolidate the response), and the most employed regimen is usually R-CHOP (Rituximab, Cyclophosphamide, Vincristine, and Prednisone), so denying the realization of the “precision medicine” that all clinicians hope to do. Nevertheless, some attempts of using different schemes for different IPI categories have been done, with opposite results: the Danish group adopted R-CHOEP-14 (R-CHOP plus etoposide every 14 days) instead of R-CHOP-14 for aggressive diffuse large B-cell lymphomas, improving prognosis (4 years OS, 75 vs. 62%) (8). Contrariwise, the adoption of dose-escalated sequential high-dose therapy and rituximab (R-MegaCHOEP) followed by autologous stem cell transplantation in high-risk DLBCL patients aged <60 years was not superior to the conventional R-CHOEP while was associated with more toxic effects (9).

On these premises, in the last 20 years many groups around the world focused their researches on the evaluation of the “minimal residual disease” (MRD), in order to tempt to identify a disease recurrence before the time when the “conventional” laboratory or imaging tools are able to demonstrate it. Indeed, in the acute promyelocytic leukemia, the treatment based on the molecular instead of the clinical relapse has been successful (10), and also in chronic myeloid leukemia the guidelines state the switch to another tyrosine kinase inhibitor when the *BCR-ABL1/ABL1* ratio does not meet the optimal value at a fixed timpe-point, so reducing the risk of transforming it into acute leukemia (11).

Thus, in 2019, MRD remains a “hot topic:” a survey performed by the Italian Society of Experimental Hematology (SIES), involving 40 Italian hematological centers, showed that for 16% of them the MRD in Non-Hodgkin's lymphomas (NHLs) represented the first topic of research (http://www.siesonline.it/survey-ricerca/). Moreover, a network aimed to detect MRD in FL, MCL, and acute lymphoblastic leukemia has been established in 2001; today, this “EURO MRD Consortium” (http://euromrd.org/usr/pub/pub.php), includes 57 laboratories across 23 countries in Europe, Israel, Singapore, Japan, Australia, USA, and South America. In the context of this Consortium, the afferent laboratories realized the standardization of the polymerase chain reaction (PCR), according to the BIOMED-1 (12) and BIOMED-2 (13) protocols. More recently, the EURO CLONALITY NGS group (https://www.euroclonalityngs.org/usr/pub/pub.php) started to harmonize methodologies necessary for analyzing the immunoglobulin heavy chain (*IGH*) and T-cell receptor (*TCR*) rearrangements by using the “Next Generation Sequencing” (NGS) tools.

This manuscript will review the history of the MRD in NHLs (see Table 1), focusing on the new frontiers of the molecular MRD detection, advantages and disadvantages of the different techniques (see Table 2) and how MRD could lead in a next future to drive the therapeutic strategies.

**Table 1:** The history of MRD in NHLs.

**Item**	**Method**	**Findings**	**References**
Autologous transplantation	PCR, RQ-PCR	Contamination of graft is predictive of relapse in FL MRD after ASCT is prognostic	(14–26)
Chemo-immunotherapy and maintenance	PCR, RQ-PCR, ddPCR	MRD represents an additional value in respect of clinical response and PET negativity in FL, MCL DLBCL and ALCL R-bendamustine has got the highest MRD clearance power in FL Monoclonal antibodies in maintenance sustain the MRD negativity and MRD after maintenance is prognostic in FL, also in stage I/II	(25, 27–42)
New therapies	PCR, RQ-PCR, ddPCR	Obinutuzumab is a promising monoclonal antibody in localized FL It is possible to design new PET/MRD-driven trials MRD can be used as platform for CART	(43, 44)
Compartments	RQ-PCR, ddPCR, NGS	MRD assessed in plasma is probably more predictive than bone marrow or peripheral blood, at least in DLBCL Cell-free DNA is a promising target Mutational MRD can be also a promising prognostic tool, at least in DLBCL	(45–49)

**Table 2:** Summary of molecular techniques used for MRD assessment in NHL.

**Method**	**Sensitivity**	**Target**	**Pros**	**Cons**
Qualitative PCR	10^−5^	IGH, TCR, BCL1/IGH, BCL2/IGH	Sensitive Standardized	Not quantitation Nested reaction
Quantitative PCR	10^−4^/10^−5^	IGH, TCR, BCL1/IGH, BCL2/IGH	Sensitive Quantitative Standardized	Need of standard reference curve for quantitation
Digital PCR	10^−5^	IGH, TCR, BCL1/IGH, BCL2/IGH, B-RAF V600E, MYD88 L265P	Sensitive Quantitative Useful also for mutations Possibility of multiplex No need of standard curve for quantitation Rapid	No standardization platform-specific
NGS	10^−4/^10^−5^	IGH, TCR, Mutations	Quantitative Multitasking	No standardization Higher costs More laborious

Under the definition of “MRD” are included all methods able to measure a disease when the “conventional” tools are not able to detect it. Technologies used to evaluate and measure MRD can be nowadays divided into three main categories: (1) those coming from the molecular biology, (2) the flow cytometry, and (3) the imaging.

Focusing on the molecular techniques, for performing MRD analysis it is necessary to identify *ab initio* a molecular marker that will be followed during or after treatment: in general, for B-cell lymphomas, rearrangements of *IGH* or of immunoglobulin light chains (kappa or lambda, *IGk, IG*λ) (50, 51) can be used; for T-cell lymphoma it is available the rearrangement of T-cell receptor (*TCR*) (52); for FL, the *BCL2/IGH* rearrangement (53, 54); for MCL, the *BCL1/IGH* rearrangement (55, 56); for hairy cell leukemia (HCL), the *B-RAF V600E* mutation (57), and for Waldenstrom's macroglobulinemia (WM) the *MYD88 L265P* mutation (58).

About flow cytometry, CD20 and CD19 characterize all B-cell lymphomas, while CD3, CD4, and CD8 are characteristic of T-cell histotypes; CD11c is typical for HCL, CD30 is detected in some T-cell lymphomas, but, differently from the chronic lymphocytic leukemia (CLL) (59, 60) or multiple myeloma (61, 62), where specific antibodies combinations and guidelines for MRD detection are available, in NHLs the role of flow cytometry in the MRD scenario is not well-established, perhaps because there is not a strict correlation between flow cytometry and microscope or molecular biology. Some papers reported that the correlation between flow cytometry and molecular results characterizes 80–85% of cases, with 10% of samples defined as MRD-positive by PCR but negative by flow cytometry and about the half of cases negative by microscope but positive on flow cytometry. In contrast, there are about 15% of the cases scored as positive by microscope that result PCR-negative, that could be the result of a patched infiltration of the bone marrow. Finally, we have to consider that sensitivities are different (1 × 10^−5^ for PCR, 1 × 10^−4^/10^−5^ for flow cytometry, 1 × 10^−2^ for microscope), and also this aspect could explain the observed discordances among the different techniques (63–65). Use of flow cytometry as tool for assessing MRD relies on the identification of a disease-specific aberrant immunophenotype. In respect of the molecular techniques, flow cytometry is performable in a shorter time, with relatively low costs; nevertheless, it requires viable cells that are to be analyzed within 48 h from sampling. Differently from CLL, in the other types of NHLs the immunophenotype is not really characteristic, and so flow cytometry is not widely employed for detecting MRD (66), except for HCL, where the combined expression of CD11c, CD103, CD123, and DBA44 allows to identify the neoplastic clone that can be then used during treatment and follow-up (67). A eight color flow cytometry has been applied in a series of 34 HCL patients and compared to quantitative PCR: sensitivities were comparable, reaching in both cases 1 × 10^−4^; both techniques significantly predicted relapse after 2CdA, so sustaining the possibility of using flow cytometry as a valid tool for testing MRD in HCL (68).

About the imaging, ultrasonography, Computed Tomography (CT), radiography, and Magnetic Resonance (MR) are usually employed for assessing clinical outcome; the positron emission tomography (PET), using fuoro-deoxi-glucose as tracer, has been reported to play an independent and relevant prognostic role in NHLs. In FL, patients still PET-positive after chemo-immunotherapy present a significant higher risk of relapse (69, 70); recently, a great interest has been given to the “total metabolic tumor volume” (TMTV), that refers to the metabolically active volume of the tumor measured by PET, and it has been shown to be an useful predicting tool. It has been reported that a TMTV higher than 510 cm^3^ would be associated with a markedly inferior survival (5 years PFS, 33 vs. 65%), independently from the FLIPI2 score (71). Analogously, the French group showed that TMTV significantly correlated with molecular MRD assessed on circulating tumor cells (CTCs) and on cell-free DNA (cfDNA): 4 years PFS was lower in patients with TMTV > 510 cm^3^, CTCs > 0.0018 cells, or cfDNA > 2,550 equivalent-genome/mL. In multivariate analysis, cfDNA and TMTV both remained predictive of outcome (72). Finally, also the reduction of TMTV at the middle of induction seems to be predictive of long-term outcome, with a reduction >66% being associated with a better prognosis in a series of 48 FL patients (73).

Analogously, in DLBCL, the interim PET was able to identify patients with worse outcome and to show that half of patients have to review their not effective therapeutic strategy (74, 75).

## MRD and Molecular Techniques

It is obvious that an excellent MRD approach has to be characterized by a high sensitivity level reached by optimizing the available technologies after complex procedures of standardization and harmonization.

MRD molecular techniques have been developed from the “classic” qualitative and quantitative PCR approaches to the new digital droplet PCR (ddPCR) and the NGS tools.

Qualitative PCR was the pioneer molecular approach implemented for NHLs in 1990: in particular, in FL, the translocation t(14;18) juxtaposes chromosome 14 and 18, posing the *BCL2* gene under the control of the heavy chain immunoglobulin enhancer, with an increased *BCL2* anti-apoptotic activity. Although this genetic event occurs in more than 80% of FL patients, as documented by the Fluorescent *in situ* Hybridization (FISH), *BCL2/JH* rearrangement is detectable by PCR only in 55–60% of FL patients (76). Three classes of *BCL2/IGH* rearrangements have been already described: the most frequent, that occurs in about half of cases, is the **M**ajor **B**reakpoint **R**egion (MBR), then the **M**inor **C**luster **R**egion (mcr), and lately the so called “minor” or “rare” rearrangements, located at the 3′ end of the MBR (3′ MBR) or at 5′ end of the mcr locus (5′ mcr), that occur in about 5% of cases (77). Qualitative and quantitative PCR approaches are available for all these *BCL2-IGH* rearrangements and they are used for marker screening at diagnosis and then for MRD evaluation. In particular, qualitative assays are performed by a nested PCR using broad and internal primers annealing both the chromosome 14 and 18. Similarly, the quantification of *BCL2/IGH* rearrangements localizes the primers and the probe in a small region very thigh to the breakpoint sites.

MCL is characterized by the translocation between chromosome 11 and 14, that once again juxtaposes the *BCL1* gene under the control of the *IGH* enhancer, thus increasing the *BCL1* pro-proliferating action. As for FL, also in MCL, where the translocation is detected by FISH in 70% of patients, the most frequent breakpoint is namely **M**ajor **T**ranslocation **C**luster (MTC), detectable by both qualitative (PCR) and quantitative PCR (RQ-PCR) in about 30% of patients (78). In MCL, *IGH* clonal rearrangements represent another molecular marker for MRD analysis in more than 60% of patients; this is due to the histological origin of these clonal cells from the mantle region of lymph nodes, where no somatic hypermutations of the variable region of immunoglobulins (*VH*) are still happen (79).

**Figure 1:**
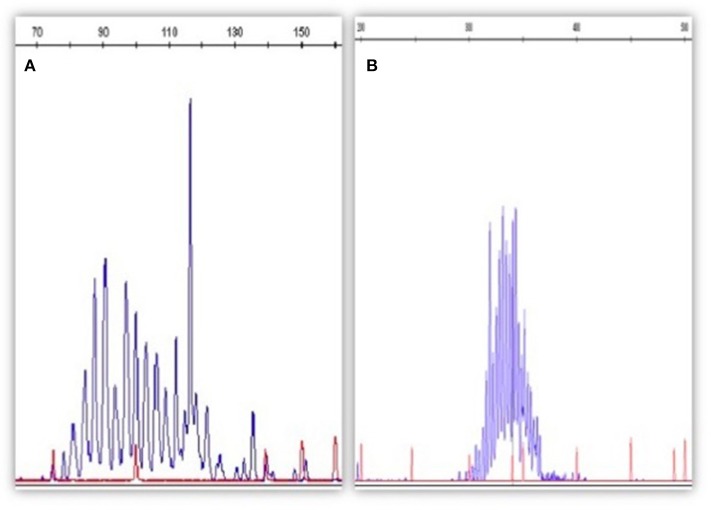
The figure depicts an example of qualitative PCR for *IGH* rearrangement (according to the BIOMED strategy). In **(A)** is represented a B clone in a polyclonal context (MRD-positive); in **(B)** the *IGH* rearrangement appears as polyclonal (MRD-negative). Qualitative PCR has been performed by Genescan method (fluorescent PCR followed by the capillary electrophoresis on a automatic DNA sequencer).

Qualitative PCR can be performed using fluorescent primers in order to run the PCR product on a DNA sequencer (capillary electrophoresis); in this way, the clonal or polyclonal pattern will be more visible and easier to be read (Figure 1).

**Figure 2:**
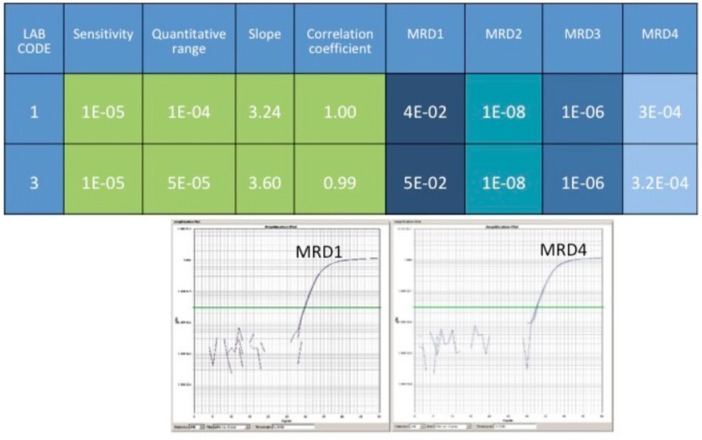
The figure represents a comparison of results coming from two different labs. RQ-PCR for *BCL2/JH* rearrangement has been performed. As reported, the sensitivity of the test reached 1 × 10^−5^, and the quantitative ranges 1 × 10^−4^ and 5 × 10^−5^, respectively. The tested sample, MRD-positive at the first follow-up, became MRD-negative at the second control, then positive but not quantifiable (at the limit of detection), and finally MRD-positive again. In the bottom panel, are represented the real plots from MRD1 and MRD4.

For RQ-PCR assays, *IGH* rearrangements are screened using single or multiplex PCR approaches followed by Sanger sequencing allowing to obtain the complete *IGH* sequence revealing the patient-specific insertions (N) among the V, D, J recombined regions. N insertions are required to set MRD quantification by the “allele specific oligonucleotide” PCR (ASO-PCR) (77). *BCL2/IGH, BCL1/IGH*, and *IGH* rearrangements detected by RQ-PCR are actually defined as the gold standard approaches for MRD evaluation because of the reached sensitivity levels, detecting up to 1 clonal cell among 100,000 analyzed (1 × 10^−5^) (76) (Figure 2). Nevertheless, RQ-PCR still has some potential technical and biological pitfalls that affect its use in NHLs: for example, the detection power of *BCL2/JH* rearrangement in only 60% of FL cases reveal a group of patients in which it is not possible to monitor MRD by this molecular technique. Moreover, for the *IGH*-positive cases, the set of the standard curve necessary for MRD quantification could be affected by the entity of tissue tumor infiltration at diagnosis, so making the quantitation not always accurate. The introduction of new methodologies, such as ddPCR and NGS, could overcome these limitations of the RQ-PCR.

**Figure 3:**
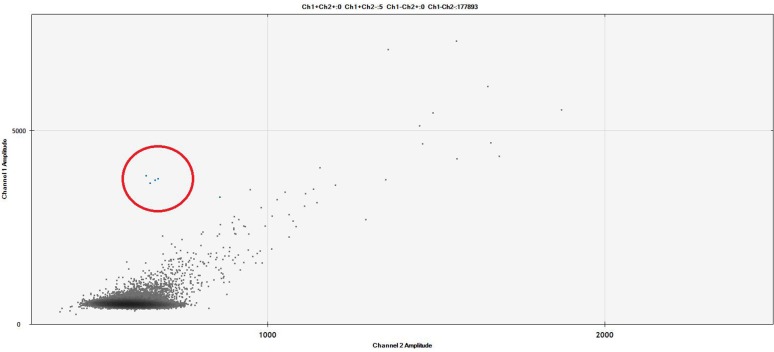
The figure represents a ddPCR Fluorescent Amplitude Plot. The droplets contained into the red circle correspond to 1 × 10^−4^ BCL2/JH-positive cell line (limit of detection). The results were analyzed on the base of FAM fluorescence *BCL2/JH*-linked (Y-axis: channel 1) and corrected by the unspecific background fluorescence (X-axis: channel 2). The lines identified the threshold amplitudes of positive vs. negative signals (ch1: 3000 RFU), and specific vs. unspecific signals (ch2: 1000 RFU). Experimental session details: the experimental session was set up using three replicates of unknown samples (plasma cfDNA extracted by QIAamp Circulating Nucleic Acid Kit – Qiagen, Milan, Italy), six replicates of negative pooled samples, two replicate of diluted positive cell line (DOHH2) and two replicate of a No Target Control (NTC) sample. The cfDNA sample was tested also for housekeeping gene. All replicates reached >10,000 droplets, the cut off for defined as technically valid a ddPCR analysis. Patient was MRD-negative.

**Digital droplet PCR** technology is based on the sample partition into many thousand droplets, so that one single DNA copy is introduced into a single droplet. After an end-point amplification phase, the appropriate software counts and quantifies the numbers of droplets containing a positive PCR product. This technology does not require the reference standard curve because, according to the Poisson's statistics, the final quantitative results take into account that it would be possible that in some droplets do not enter any DNA molecule or that in other droplets would be co-present two or more DNA copies (80) (Figure 3). ddPCR was compared to the classic PCR in NHLs and other B lymphoprolipherative diseases: Drandi and coworkers showed superimposable results, with a higher sensitivity level for ddPCR, that allowed to find the molecular marker also in samples with very low tumor infiltration. ddPCR is not only a sensitive and accurate technique, but it has got the great advantage of avoiding the standard curve, with consequent reduction of the contamination risk (81). The increasingly relevant role of ddPCR is clearly shown by the many fields of its application in hematology, such as detection of *JAK2 V617F* mutation in chronic Philadelphia-negative myeloproliferative neoplasias (82), *PML-RARa* rearrangement in acute promyelocytic leukemia (83), *BCR-ABL1* rearrangement in chronic myeloid leukemia (84), *B-RAF V600E* mutation in HCL (85), *IGH* rearrangement in acute lymphoblastic leukemia (86) and *MYD88 L265P* mutation in Waldenstrom's Macroglobulinemia (MW) (87). In WM, it has been recently shown that ddPCR reached a sensitivity of 5 × 10^−5^, 1.5 log higher than that offered by ASO-PCR. In a series of 148 patients affected by WM, lymphoplasmacytic lymphoma or IgM monoclonal gammopathy of undetermined significance (MGUS), 95% of cases showed the *MYD88* mutation; MRD tested on plasma and urines was more sensitive than the assessment on bone marrow or on the peripheral blood mononuclear cells (87).

Another application of ddPCR in the context of MRD is the assessment of *B-RAF V600E* mutation in HCL: the sensitivity of this test resulted higher than that offered by RQ-PCR (5 × 10^−5^ vs. 2.5 × 10^−4^), with the same specificity. After treatment with Rituximab and 2-chloro-deoxi-adenosine (2CdA), ddPCR showed that 33% of subjects in complete remission (CR) were still MRD-positive, vs. 28% scored as still MRD-positive by RQ-PCR and 11% by the qualitative PCR for *IGH* rearrangement. After 12 months of follow-up, ddPCR still detected *B-RAF* mutation in 15% of cases otherwise defined as MRD-negative, and only this technique showed a prognostic role in terms of long-term PFS (85). After these promising results, the need of ddPCR standardization has become necessary, and now it is ongoing within the EURO MRD Consortium.

**Figure 4:**
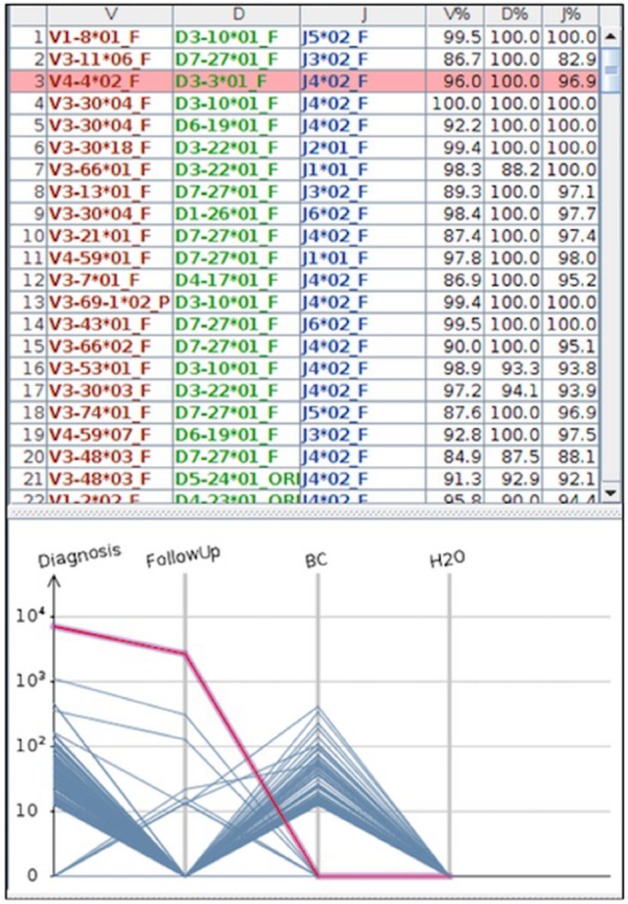
The figure represents a case where MRD was tested by NGS. In the part above the *IGH* clones found by the HashClone software [(91) BMC Bioninformatics], are detailed; in the bottom part the *IGH* frequencies clones describing MRD monitoring in diagnostic and follow-up samples are depicted. As reported, MRD at the follow-up became negative; BC = positive control; H_2_O = negative control (water). MRD was performed using primer annealing *IGH* framework region 1 and JH loci, and MiSeq Illumina platform was used for the sequencing.

Beside ddPCR, **NGS** technologies are currently developing to increase the portfolio of molecular techniques useful for MRD detection. In particular, NGS, either “mutational” or “amplicon-based,” is now object of interest, both for the broad spectrum of targets to be investigated and also for the very deep analysis levels (defined as variant allele frequencies and coverage) reached compared to the classic sequencing methods (88). Recently, a “capture-based” protocol covering the coding V, D, and J genes of the *IGH* loci was designed; applied to B- and T-cell disorders, this approach showed that using capture probes against V, D, and J regions (with additional switch regions), clonal rearrangements were detectable in 21 out of the 24 tested patients. The amplified region encompassed 180 kb and the average depth of sequencing was 322x. Of the 3 failed samples, in two cases DNAs were degraded or of low-quantity and in one there was a technical error. This work, realized in the context of the EURO CLONALITY MRD Consortium, is the final demonstration of the real possibility of including NGS in the techniques today available for MRD detection (89). The sensitivity of NGS is still matter of debate: several authors, especially in the myeloma setting, reported values of 1 × 10^−6^/10^−7^ (90): it has to be considered that these values are achievable only if more than 5 μg of tumor DNA are employed, and in a specific “closed” system offered by some companies (such as Adaptive, USA). Consequently, outside this specific setting, the standardization of NGS is still not a reality. Nevertheless, within the EURO MRD Consortium, the EURO CLONALITY NGS group (https://www.euroclonalityngs.org/usr/pub/pub.php) is working to standardize the wet lab procedures and the bio-informatic tools able to introduce NGS in the routine MRD workflow. Today, commercially kits are also available that can make the use of NGS and the interpretation of its data easier for each laboratory (Figure 4).

## MRD in Follicular Lymphoma

In the middle of 90's, before the “Rituximab era,” the majority of FL patients rapidly relapsed, and frequently underwent to autologous stem cell transplantation (ASCT) that offered 90% of CRs and long-term survival to more than 50% of patients (14, 15). Thus, the first studies concerning MRD in FL were designed to answer two questions: (1) if the contamination of the graft by neoplastic cells could condition the further outcome, and (2) if the MRD status after ASCT could have a prognostic role. About the first question, different studies showed that contaminated harvests could play a negative impact on progression-free-survival (PFS) and overall survival (OS) (16). When Rituximab entered into the clinical practice and RQ-PCR became available, it was clear that Rituximab was able to purge the apheretic products when used either *ex vivo* or *in vivo*, and that the infusion of grafts uncontaminated or with a lower degree of contamination was associated to a better outcome (17). About the role of MRD after ASCT, it was clear that there were three different categories of patients: (1) those who, initially PCR-positive, reached the MRD negativity after ASCT; (2) those always MRD-positive; (3) an intermediate category, including cases initially MRD-positive who reached the MRD negativity after transplantation or during follow-up or patients who were MRD-negative after induction but became then MRD-positive (the so called “mixed” group); for this latest group, the prognosis resulted intermediate between the first and the second cohort (18, 19).

After the introduction of Rituximab in the clinical practice, it was immediately clear that this monoclonal antibody was able to increase the number of MRD-negative cases: in a pivotal study, 74% of patients receiving Rituximab converted to the MRD negativity and this status was associated with a better clinical outcome (failure free from recurrence, 57 vs. 20% for those patients who never achieved or lost the MRD negativity) (27). Thus, after the advent of Rituximab, the focus of the molecular studies assessing MRD in NHLs translated from the ASCT to the chemo-immunotherapy: in 2010, the European Organization for Research and Treatment of Cancer (EORTC) assessed MRD in 465 relapsed FL patients randomized to receive Cyclophosphamide, Doxorubicine, Vincristine, and Prednisone (CHOP) or R-CHOP (CHOP plus Rituximab) as induction and then Rituximab or not as maintenance. The authors did not find any prognostic role for MRD (evaluated as persistence of *BCL2/IGH* rearrangement) when it was evaluated just after induction; in contrast, the MRD status was able to distinguish two groups with different risk or relapse when assessed after maintenance, so confirming the positive impact of maintenance with Rituximab also in terms of the molecular disease clearance (28). The fact that authors did not find a clear prognostic impact of the MRD precociously assessed could depend on the chosen evaluation timing: indeed, the MRD was evaluated by 2 months from the end of treatment, when Rituximab is still in the circulation and could probably falsely clear the molecular marker, especially from the peripheral blood (PB). Analogous “negative” results were obtained when MRD was assessed in strict proximity of the end of chemo-immunotherapy also in another big trial where R-CHOP was compared to Rituximab, Cyclophosphamide, Vincristine, and Prednisone (R-CVP) and Rituximab, Fludarabine, and Mitoxantrone (R-FM) in 534 advanced FL patients: the Foll05 study. In this Italian multicenter trial, *BCL2/IGH* was detected at diagnosis in half of cases; after induction, 70% of patients became MRD-negative, but the MRD status evaluated in proximity of the end of induction was not predictive of PFS. On the contrary, MRD negativity had a positive impact on PFS when assessed at 12 or 24 months of follow-up (3 years PSF, 72% for cases in CR and MRD-negative vs. 32% for those in CR but MRD-positive) (29). Differently from that observed in the EORTC trial (28), in the Italian study the “molecular burden” measured at diagnosis well-correlated with the quality of clinical response and PFS: in fact, patients with a higher initial molecular burden (cut off 1 × 10^−4^) achieved CR in a percentage inferior to that observed in cases with lower “molecular burden” and presented a shorter PFS (3 years PFS, 58% for cases with “high” vs. 92% for those with “low” molecular burden) (29). These results have been recently also confirmed after a longer follow-up (7 years PFS, 48% for cases with “high” vs. 74% for patients with “low” molecular burden) (FIL MRD network, data presented at the Lugano ICM 2019 meeting).

To the same conclusions went also another trial, the ML17638, where the role of Rituximab in maintenance was assessed in 227 elderly FL patients after a brief chemo-immunotherapy. Also this study confirmed the prognostic role of MRD: patients MRD-negative had a 3 years PFS of 72 vs. 39% for those who were still MRD-positive after 8 months. Moreover, 3 years PFS was 77% for cases in CR/MRD-negative, 59% for patients in PR/MRD-negative, 45% for those in CR but MRD-positive, and only 5% for subjects in PR and MRD-positive, so showing that in FL MRD really can represent an adjunctive value to the clinical response (30).

In addition to Rituximab, another anti-CD20 antibody, the ^90^Yttrium-Ibritumomab-Tiuxetan, revealed to be efficacious as consolidation in FL (31). In the Zeus trial, 50 untreated FL patients with a low tumor burden received a single treatment with this radiolabelled antibody: 86% of them achieved CR, with 3 years PFS and OS of 63 and 90%, respectively. In this study, it was evident that ^90^Yttrium-Ibritumomab-Tiuxetan was able to perform a good clearance of MRD, because the molecular burden decreased from an initial median value of 2,342 to 4.6 copies/mL at the end of treatment. Moreover, this reduction positively impacted on PFS (30 months PFS, 80% for MRD-negative vs. 46% for MRD-positive cases) (32).

Also the role of Bendamustine in terms of MRD clearance has been investigated after demonstration by the German group that PFS was significantly advantageous for patients receiving R-bendamustine in respect of those treated with the R-CHOP (33, 34). At the ASH meeting held in 2018, Dr. Pott presented the molecular results from the Gallium trial, where Obinutuzumab was compared to Rituximab in a series of 1,202 advanced FL patients (35). Obinutuzumab represents a second-generation of anti-CD20 monoclonal antibodies, designed for overcoming the resistance to Rituximab and for exerting a more effective action in B-cell lymphoproliferative diseases. It is a monoclonal antibody of the IgG1 subclass derived by humanization of the parental B-Ly1 mouse antibody by recombinant DNA technology, carrying a glyco-engineered Fc portion, with higher affinity for FcgRIIIa receptors on immune effector cells, and a stronger antigen binding activity. Moreover, while Rituximab stabilizes CD20 on the lipid rafts, Obinutuzumab leave CD20 distributed across the surface of the B cell; this difference results in a lower complement-dependent cytotoxicity (CDCC) but greater antibody-dependent cellular cytotoxicity (ADCC) and direct cell death (DCD) in comparison to Rituximab (92). Obinutuzumab is now indicated in combination with chlorambucile as first-line treatment for CLL, and in FL, in untreated advanced-stage patients or in refractory/progressed cases in association with bendamustine and as maintenance (for details, https://www.gazyva.com/hcp/flfl/efficacy/pfs.html).

The Gallium trial clearly showed that the Obinutuzumab-based chemotherapy reduced the probability of progression, relapse or death up to 30%. Interestingly, for the first time, MRD was assessed not only at the end, but also at the middle of induction: with a median follow-up of 57 months, cases MRD-positive at the middle of induction presented a probability of remaining without progression that was 22% lower in comparison to patients who achieved MRD negativity at the same time-point. At the end of induction, 92.6% of patients treated with Obinutuzumab and 85.2% in the Rituximab arm became MRD-negative; the difference in terms of MRD clearance between patients in the obinutuzumab arm in respect of the rituximab arm was still evident also after maintenance. Moreover, PFS was longer for patients already MRD-negative at the middle of induction (“early responders”) in respect of the “late responders” (those MRD-positive at the mid of induction but MRD-negative at the end of treatment) and of the “always MRD-positive” cases (4 years-PFS, 80% for the “early responders” vs. 60% for the “late responders” vs. 30% for “always MRD-positive” cases) (92). The Gallium trial reported also that MRD negativity significantly impacted not only on PFS but also on OS (hazard ratio, 0.35) (36) and demonstrated the superiority of the combination of Obinutuzumab with Bendamustine in respect of with CHOP or CVP. Indeed, when combined with CHOP or CVP, Obinutuzumab increased the MRD negative cases of 13–15% in comparison to Rituximab, but, when added to Bendamustine, this advantage decreased to only 3%, thus supporting the hypothesis that Bendamustine would be more effective in terms of MRD clearance (92).

To the same conclusion went also another small and retrospective study assessing MRD in a series of 48 FL patients receiving R-Bendamustine as first-line therapy: 93% of patients became MRD-negative after treatment, and the MRD-negativity at 6 months played a favorable prognostic impact on the 30 months PFS (80% for MRD-negative cases vs. 46% for MRD-positive ones). The median clearance of molecular disease was 3 logs, a value higher than that observed in the Foll05 or Zeus trials (37).

## MRD in Mantle Cell Lymphoma

As above reported for FL, also in MCL the MRD was initially employed to further demonstrate the efficacy of the high-dose consolidation after several induction strategies: 29 patients treated with chemotherapy and ASCT were assessed by RQ-PCR for *IGH* rearrangement. After ASCT, 52% achieved MRD negativity, and the molecular remission was strongly predictive for an improved outcome, with a median PFS of 92 months in the MRD-negative group compared with 21 months in the MRD-positive group and a median OS of 44 months in the MRD-positive group vs. not reached in the MRD-negative group (20).

Other trials reported a positive effect of ASCT on the MRD clearance: in the Lyma trial, ASCT increased the MRD-negativity rate from 66 to 82%, and in the MCL3 Nordic study from 53 to 83%; in these studies, the MRD positivity after ASCT increased the risk of relapse up to 4 folds (21). The European MCL Network conducted a phase-3 randomized trial where patients aged <65 years were randomized to receive either six courses of R-CHOP followed by ASCT or six courses of alternating R-CHOP or R-DHAP, then high-dose cytarabine and ASCT. After induction, 61% of patients were MRD-negative in the group receiving cytarabine vs. 26% of cases receiving conventional treatment, and the survival was longer in the group receiving cytarabine than in the standard one (freedom from failure = 9.1 vs. 3.9 years). After ASCT, the proportion of MRD-negative patients were higher in the cytarabine than in the control group (85 vs. 68% in PB, and 79 vs. 59% in BM). ASCT was able to increase the proportion of MRD-negative patients more in the control group (from 37 to 60%) than in the cytarabine group (from 70 to 85%), and the achievement of MRD negativity either after induction or after ASCT was a strong prognostic factor for PFS, independently from MIPI score, Ki-67 value or quality of clinical response (22). Moreover, the Nordic Lymphoma Group assessed MRD in 183 MCL patients who underwent ASCT by performing PCR for BCL1/JH and IGH rearrangements. Shorter PFS and OS were demonstrated for patients who were MRD positive pre- or after-ASCT (median PFS, 20 months in the MRD-positive group vs.142 months for the MRD-negative one; OS, median not reached vs. 35 months) (23). Finally, in the cohort of patients receiving ASCT in CR, the molecular status before ASCT was predictive of the outcome: the median OS for MRD-negative patients was not reached, with 82% survival at 5 years, whereas for the MRD-positive patients median OS was 3 years. The median PFS for MRD-negative patients was not reached with 75% PFS at 5 years, whereas it was 2.4 years for MRD-positive patients (24).

As previously described for FL, also in MCL the prognostic impact of MRD was analyzed in the context of the chemo-immunotherapy: 259 patients treated within two randomized trials of the European MCL Network (MCL Younger and MCL Elderly trial) were tested for MRD after induction: 56% of cases resulted MRD-negative, with a significant advantage in terms of 2 years PFS (87% for MRD-negative vs. 61% for MRD-positive cases). In these studies, the MRD status represented an additional value in respect of the clinical response, because 94% of cases in CR/MRD-negative remained disease-free at 2 years vs. 71% of patients in CR but still MRD-positive; in the group of patients in partial response (PR), 2 years PFS was 100% for those MRD-negative compared to 51% for the MRD-positive ones. Finally, once again the sustained MRD negativity was predictive of a better outcome either after ASCT in the young cohort or after maintenance in elderly patients (25).

In 2012, results from the R-HYPER-CVAD regimen (containing Cyclophosphamide, Vincristine, Doxorubicin, and Dexamethasone in courses A and Methotrexate and Cytarabine in courses B) were published: after two cycles, 50% of patients became MRD-negative; at the end of treatment, the MRD negativity rate increased to 83%; nevertheless, the MRD status did not significantly impact on the outcome, probably because of the small number of cases enrolled (38).

At the ASH held in 2018 were presented results from the FIL MCL0208 trial, a prospective, randomized study that compared maintenance with Lenalidomide vs. observation after an intensive chemo-immunotherapeutic regimen and ASCT in 300 MCL young patients. A molecular marker (both *BCL1/JH* and *IGH* rearrangements) was found in 83% of patients, and the MRD-negativity was achieved in 78% of patients after the high-dose chemotherapy and in 79% after ASCT. MRD positivity at every time-point showed a 2-folds higher risk of relapse or death: 3 years-PFS was 53% for patients MRD-positive vs. 66% for those MRD-negative, and the presence of at least two consecutive MRD-negative results conferred a significantly reduced risk of relapse. Finally, in this study the RQ-PCR offered a better risk stratification than the qualitative PCR (39).

In the Cancer and Leukemia Group B (CALGB) 50403 trial, presented at the same ASH meeting, MRD was assessed after induction, ASCT and consolidation or maintenance with Bortezomib. The 8-years PFS was increased from 50% of the historical control to 58% in the cohort receiving Bortezomib as consolidation and to 77% for patients receiving maintenance. The outcome was significantly advantageous for MRD-negative cases (8 years PFS, 80% for MRD-negative vs. 43% for MRD-positive cases) (26).

In conclusion, in MCL all studies are concordant in the sustaining the prognostic role of MRD.

## MRD in T-cell Lymphomas

Not many data about MRD in T-cell lymphomas have been produced, probably for the poor prognosis of these malignancies. In particular, the few available results concern the anaplastic large cell lymphomas (ALCL) characterized, in about 75% of cases, by the expression of the anaplastic lymphoma kinase (ALK) and of cytokine receptor CD30 (93). The translocation between chromosome 2 and 5 gives origin to the *NPM-ALK* fusion gene, that can be used as molecular marker for MRD assessment: the Czech group reported that the monitoring of this fusion gene by RQ-PCR was predictive of relapse, that was predicted by the increase of the transcript of 0.5 logs (94). More recently, other two groups analyzed the prognostic role of the *NPM-ALK* fusion gene in 180 childhood patients. The molecular marker was found in 57% of cases, and its presence at diagnosis was predictive of a higher relapse rate and shorter PFS and OS. After treatment, half of patients became MRD-negative; the probability of relapse for cases PCR-positive at diagnosis/MRD-positive after treatment was significantly higher than that observed in cases PCR-positive/MRD-negative (81 vs. 31%) or in patients without initial molecular marker (15%). Five-year survival of PCR-negative and PCR-positive/MRD-negative patients was 91 and 92%, compared with 65% of cases PCR-positive/MRD-positive, so demonstrating the negative impact either of the molecular marker at diagnosis or of the MRD in this kind of malignancy (40).

## MRD in Aggressive Lymphomas

Definition of Diffuse Large B Cell Lymphoma (DLBCL) includes several different entities: the “Activated B-cell like” (ABC) lymphoma, the “Germinal Center B-like” (GCB) and the “Primary Mediastinal” B-cell lymphoma (PMBL). Each of these subtypes is characterized by a different genomic profile, being mutations of *EZH2* more frequent in the GCB, mutations of *MYD88* or *INK4A-ARF* more often detected in ABC, and mutations of *XPO1* characterizing more frequently PMBL (95). The recent availability of the new techniques, such as NGS and Nanostring, allowed to identify the histotype in a more precise way than with immunohistochemistry (96, 97); thus, once identified the patient-specific “mutational profile” at diagnosis, these mutations can be used for monitoring the “mutational MRD.” At the ASH 2018, Dr. Zhang reported that NGS, set to cover mutations of 61 genes known to be significantly associated with prognosis in DLBCL, was able to detect mutations in 70% of the transformed and in 55% of the *de novo* DLBCL, and that there was a significant correlation between the mutation variant allele frequency (VAF) during follow-up and the outcome, with a longer PFS observed in NGS-negative cases (98). Classically, at diagnosis mutational tests are performed on DNA extracted by the neoplastic tissue or bone marrow; nevertheless, DLBCL infiltrates bone marrow quite rarely, and when patients well-respond to treatment no further masses are available for obtaining tumor DNA. Consequently, many researchers started to use the plasma as source of tumor DNA, either by extracting it as cell-free DNA (cfDNA) or from the circulating exosomes (45). The first paper that clearly demonstrated that plasma would be the best compartment for assessing MRD in aggressive lymphomas was published in 2015: the *IGH* and *IGK* clonalities were tested by the LymphoSIGHT technology in 105 tumor samples, and in 83% of them it was possible to find a molecular marker. Interestingly, a higher percentage of cases showed a molecular marker in plasma instead of in the peripheral blood mononuclear cells, and the molecular disease resulted twice higher in the plasma. At the time of progression/relapse, all patients were MRD-positive in the plasma while only 30% of them showed tumor cells circulating in the peripheral blood (46). More recently, the same group reported a strict correlation between the cfDNA levels and response to therapy: in patients where cfDNA levels decreased 2 logs after one cycle and 2.5-log after two cycles of chemo-immunotherapy, the EFS was significantly longer than that observed in cases with a lower molecular disease reduction. In multivariable analysis, including IPI and interim PET, MRD still retained its independent prognostic value (47).

In line with these results, our group recently showed the superiority of cfDNA in respect of circulating cells as source of tumor DNA: indeed, during the surgery for a kidney explant, a DLBCL, not detectable by CT just before procedure, was found in the donor. After 3 months, the *IGH* clone characterizing the donor's lymphoma was detected on cfDNA in the plasma of recipient, while DNA extracted from bone marrow and peripheral blood mononuclear cells was polyclonal, and he was PET/CT-negative. After further 3 months, the recipient developed an abdominal DLBCL carrying the same *IGH* rearrangement of the donor (99), thus supporting the idea that in DLBCL the plasmatic compartment is really predictive and suitable as tool for assessing MRD in the majority of patients, as previously reported (48).

At the same conclusion went Hossain and colleagues who for the first time assessed the MRD by cfDNA in 6 patients receiving the anti-CD19 chimeric antigen receptor (CAR) T cell therapy (CAR-T) for relapsed/refractory DLBCL. The “molecular” MRD after day +28 was compared to the MRD assessed by PET: in four out of five cases the increased values of cfDNA preceded progression before PET, and all progressing patients had increasing cfDNA when PET confirmed the clinical progression, thus supporting the idea that the MRD when assessed in the plasma could be the most important predictive tool of the DLBCL patients' outcome (49).

From the technical point of view, the problems of adopting molecular techniques for testing B-cell clonality are different: (1) it is possible that some patients could have a unproductive *IGH* rearrangement or a hypermutation of the variable region of the heavy chain of immunoglobulins (VH) that makes impossible the right binding of primers; (2) the DNA extracted from plasma could be not enough for molecular analysis; (3) a clone different from the original one can be appear that would be responsible for progression of disease; this eventual clone is not detectable by ASO-PCR set for the specific initial neoplastic clone. In this context, NGS could be the most appropriate technique, because it allows to detect any possible B or T clone in an “unsupervised” way, so offering the real scenario of clonality at each time-point.

Finally, another innovative and promising application of the molecular biology in DLBCL is that MRD can be employed as platform for immunotherapy: we know that anti-CD19 CAR-Ts offer to relapsed/refractory DLBCL patients an overall response rate (ORR) of 70%, 50% of CRs, and 60% of probability of surviving at 12 months (100). It has been reported that immunotherapy was more effective in cases treated in PR or in CR/MRD-positive in respect of when CAR-T are infused in subjects in overt clinical relapse (ORR 100 vs. 75%) (43).

Another aggressive lymphoma where MRD has been evaluated is the Burkitt's Lymphoma (BL); Shiramizu et al. approached this topic by RQ-PCR in a series of 32 adolescent/children, finding the molecular marker (*IGH* rearrangement) in 69% of tested cases. At the end of induction, only one patient remained MRD-positive, and he further relapsed; at the end of treatment, another patient was still MRD-positive, and also this one relapsed. Authors concluded that the low number of patients enrolled in the study was too low for definitively stating that MRD is a relevant prognostic tool also in BL, ma that it was possible to find a role for MRD also in this malignancy (41). The same group assessed MRD in a series of 10 high-risk BL patients; in this subgroup, MRD was not prognostic, notwithstanding 7/10 cases were MRD-positive at the end of induction and five at the end of consolidation (42). Consequently, the role of MRD in BL still remains a matter of debate.

## MRD and Allogeneic Transplantation

In the allogeneic transplantation setting, MRD can be used not only for following the disease and predict the eventual relapse, but also for monitoring the immune reconstitution, and then to proceed with the tapering of immunosuppression or with the donor lymphocytes infusion (DLI). Differently from CLL, in other NHLs MRD after allogeneic transplantation (alloSCT) has not been extensively studied. In CLL, it has been clearly showed that the MRD-negative status after 6 or 12 months from alloSCT (detected either by flow cytometry or by PCR) was strongly predictive for the absence of clinical relapse (101). In a series of 59 patients, the authors reported only two relapses in the 32 patients who were MRD-negative, whereas six of the 11 patients remaining MRD-positive after transplant relapsed (102). Other authors confirmed these findings showing that patients who were MRD-negative at 12 months after alloSCT showed longer 2-years OS and EFS in comparison to subjects still MRD-positive (OS, 96 vs. 56%; PFS, 83 vs. 0%) (103). In an interesting work, published in 2016 by Sellner and colleagues, MRD was assessed either by ASO-PCR or by NGS in seven patients affected by T-prolymphocytic leukemia who received alemtuzumab and then allogeneic transplantation. Two out of seven patients became MRD-negative few weeks after transplant; in one of this cases, the *TCR* clonal rearrangement suddenly reappeared, followed by a clinical relapse. In the other five cases, all MRD-positive, the tapering of immunosuppression or DLI decreased MRD of more than 1 log, and *TCR* rearrangement, skewed just after transplantation, became polyclonal (104), so sustaining the possibility of using MRD after allogeneic transplantation as predictive tool.

## How MRD Could Change the Therapeutic Strategy in the Next Future

As above reported, in the last 20 years many studies demonstrated that MRD play an evident prognostic role, especially in terms of PFS, in NHLs; nevertheless, few studies included MRD as endpoint in their initial design, and also the guidelines edited by the European Society for Medical Oncology (ESMO), even if recognizing the role of MRD in NHLs, do not yet include the molecular tests in the necessary and routinary work-up of NHL patients, sustaining the need of a definitive standardization of the molecular tools (105–107).

Nevertheless, it's time that MRD could be really translated from the bench to the bedside, and in this line some MRD-based trials are now in progress: the Fondazione Italiana Linfomi (FIL) Foll12 trial (EudractCT Number 2012-003170-60) is comparing in more than 800 untreated FL patients the “standard” (R-CHOP followed by maintenance with Rituximab) to the “experimental” arm, where patients are stratified according to MRD (defined by qualitative PCR, RQ-PCR and PET). Patients who after induction are PET-positive will receive a consolidation with ^90^Yttrium-Ibritumomab-Tiuxetan; those PET-negative but MRD-positive will receive rituximab as pre-emptive therapy, and individuals both PET- and MRD-negative will not receive any maintenance.

Moreover, another Italian cooperative study is in progress, where the MRD drives treatment in early stage FL patients: in the FIL Mirò trial (Clinical Trials.gov n. NCT02710643), subjects with localized FL receive involved-field radiotherapy; after that, *BCL2/IGH*-positive cases will receive Ofatumumab to clear MRD, whereas the MRD-negative patients will start observation.

Finally, at the 2018 ASH meeting a German group presented the results from a trial employing six cycles of Obinutuzumab and Ibrutinib as induction followed by maintenance with the same combination of drugs as first-line treatment for advanced FL patients. After induction, 70% of cases became MRD-negative, and 83% were negative at the end of maintenance. Also this trial was MRD-driven, because after maintenance (at the 30th month) MRD-positive cases receive further 12 months of Ibrutinib, whereas those MRD-negative start the observation only (44).

Results from these interesting and promising MRD-based studies will be available in the next future and they probably will definitively convince the scientific community about the real possibility of exporting MRD in the clinical practice. The new molecular techniques today available and the collaboration between biologists and clinicians will allow us to finally realize the “MRD-adapted treatment approaches” that for many years remained only a dream for the majority of physicians that every day treat NHLs.

## Author Contributions

SaG, EG, and MP wrote the manuscript. SuG, EC, FG, and EG revised the part of methodologies and performed experiments in the FIL MRD NET. LI, FrM, and FoM revised the clinical parts of the paper.

### Conflict of Interest Statement

The authors declare that the research was conducted in the absence of any commercial or financial relationships that could be construed as a potential conflict of interest.
